# Perceived Injustice in Cancer Survivors: Population-Specific Cut-Off Score and Relations with Personal Factors, Symptoms and Quality of Life—A Cross-Sectional Study

**DOI:** 10.3390/jcm12185780

**Published:** 2023-09-05

**Authors:** Eva Roose, Eva Huysmans, Astrid Lahousse, Kenza Mostaqim, Lotte van Gerven, Moniek Vissers, Jo Nijs, Paul Van Wilgen, David Beckwée, Annick Timmermans, Rinske Bults, Laurence Leysen

**Affiliations:** 1 Pain in Motion Research Group (PAIN), Department of Physiotherapy, Human Physiology and Anatomy, Faculty of Physical Education & Physiotherapy, Vrije Universiteit Brussel, 1090 Brussels, Belgium; eva.huysmans@vub.be (E.H.); astrid.lucie.lahousse@vub.be (A.L.); kenza.mostaqim@vub.be (K.M.); jo.nijs@vub.be (J.N.); p.vanwilgen@transcare.nl (P.V.W.); rinske.marije.bults@vub.be (R.B.); laurence.leysen@vub.be (L.L.); 2 Rehabilitation Research Group, Department of Physiotherapy, Vrije Universiteit Brussel, Laarbeeklaan 103, 1090 Brussels, Belgium; david.beckwee@vub.be; 3 REVAL, Universiteit Hasselt, Agoralaan-gebouw A, 3590 Diepenbeek, Belgium; annick.timmermans@uhasselt.be; 4 Department of Physical Medicine and Physiotherapy, Universitair Ziekenhuis Brussel, 1090 Brussels, Belgium; 5 Research Foundation–Flanders (FWO), Leuvensesteenweg 38, 1000 Brussels, Belgium; 6 The Berekuyl Academy, Molenweg 4, 3849 Hierden, The Netherlands; lottevangerven91@gmail.com (L.v.G.); moniek_vissers@hotmail.com (M.V.); 7 Department of Health and Rehabilitation, Unit of Physiotherapy, Institute of Neuroscience and Physiology, Sahlgrenska Academy, University of Gothenburg, Huvudbyggnad Vasaparken, Universitetsplatsen 1, 41345 Gothenburg, Sweden; 8 Transcare Pain Transdisciplinary Pain Treatment Center, 9711 Groningen, The Netherlands; 9 Department of Rehabilitation Sciences and Physiotherapy, Faculty of Medicine and Health Sciences, University of Antwerp, 2610 Wilrijk, Belgium

**Keywords:** cancer survivors, cut-off, fatigue, pain, perceived injustice, quality of life

## Abstract

Fatigue and pain are the most common side effects impacting quality of life (QoL) in cancer survivors. Recent insights have shown that perceived injustice (PI) can play a substantial role in these side effects, but research on cancer survivors is scarce. Furthermore, guidelines for recognizing clinically relevant levels of PI in cancer survivors are missing. The aims of this study are to provide a clinically relevant cut-off for PI and to explore relationships between personal characteristics, symptoms, and QoL with PI. This multicenter, cross-sectional study uses the Injustice Experience Questionnaire (IEQ), Numeric Pain Rating Scale (NPRS), Patient-Specific Complaints (PSC), Multidimensional Fatigue Index (MFI), and European Organization for Research and Treatment of Cancer QoL Questionnaire-C30 (EORTC-QLQ-C30). A clinical cut-off for PI was identified based on the 75th percentile of IEQ scores. Univariate and multivariate regressions explored the relationship between PI and personal characteristics (sex, age, cancer type, treatment type), symptoms (pain intensity, fatigue), and QoL (daily activity complaints, cancer-related QoL). Cancer survivors (n = 121) were included, and a cut-off of 20 was identified. Significant indirect associations were found between chemotherapy, NPRS, PSC, MFI, and EORTC-QLQ-C30 with PI. In the multivariate model, only MFI (B = 0.205; 95% CI: 0.125–0.018) and age (B = 0.086; 95% CI: −0.191–0.285) maintained a significant association with PI.

## 1. Introduction

Currently, 1 in 5 men and 1 in 6 women will develop cancer during their lifetime, with a global burden of disease accounting for 19.3 million new cancer cases yearly [1]. Thanks to new insights and developments in cancer treatments, the survival rates of all cancers combined have increased by 70% five years after diagnosis [2]. Cancer survivors are defined as people who have been diagnosed with cancer, in whom primary treatment has been completed (with the exception of maintenance therapy), and who show no signs of active disease [3]. However, due to increased survival rates, new chronic symptoms are occurring and persisting in cancer survivors [4]. 

The biopsychosocial side effects of cancer diagnosis and treatments cannot be underestimated [5], with justice-related appraisals receiving more attention in cancer survivors [6,7,8], especially in breast cancer [9,10,11]. Sullivan et al. (2009) defined perceived injustice (PI) as: “The experience of unnecessary suffering as a result of another’s actions, or the experience of irreparable loss” [12,13] (e.g., someone who never smoked yet was diagnosed with lung cancer). An important factor in the origin of PI is emotional distress [14], which can be caused by a cancer diagnosis and medical cancer treatment. The discrepancy between expected and actual outcomes in people with PI could possibly lead to frustration, feelings of anger, or other forms of emotional distress [15]. In research, to investigate associations with PI, the Injustice Experience Questionnaire (IEQ) was used to measure PI. Unfortunately, no clinical cut-off for PI on the IEQ for cancer survivors has been identified yet. Up until now, only a clinical cut-off score of 30 has been known for the IEQ in individuals who have sustained musculoskeletal injuries [16] or 19 for people with whiplash-related symptoms according to the risk and outcome of employment status [17].

Recent systematic reviews have highlighted the inter-correlated nature of PI with pain-related outcomes [18,19], psychological outcomes [18,19,20], and quality of life [19]. PI may, thus, be important to consider during cancer survivorship. It is already known that PI mediates the relationship between pain, sleep, and fatigue in breast cancer survivors [11]. Fatigue is the most prevalent symptom experienced by cancer survivors [21] and is seen in 52% of cancer survivors [22]. Persistent cancer-related fatigue impacts the quality of life (QoL) since cancer survivors can become too fatigued to fully participate in daily living [23,24]. In non-cancer pain populations, PI also predicts adverse pain outcomes even when controlling for other pain-related psychosocial constructs, such as pain catastrophizing and fear of movement [13,25,26,27]. Increased pain in breast cancer survivors has been seen, with more PI and decreased QoL [9]. Chronic pain is highly prevalent among cancer survivors (33%) [28] and impacts their health-related QoL [29,30,31,32,33,34]. This prevents the resumption of pre-diagnosis activities, leading to a high socio-economic burden [29,30,31,32,33,34]. Taken together, PI appears to have a negative influence on various key rehabilitation factors, making it an important factor to consider in cancer survivors. However, so far, it is still unclear which personal characteristics (e.g., sex, age, type of cancer, treatment type) in cancer survivors are associated with PI. 

Given the already established importance of PI in breast cancer survivors [9,10], the role of PI in cancer survivors requires further research, and it is necessary to define a clinical population-specific cut-off point to identify clinically relevant PI. Therefore, the aims of this cross-sectional study are to provide a clinically relevant cut-off score for PI in cancer survivors and to explore the relationship between personal characteristics (sex, age, type of cancer, treatment type), symptoms (pain intensity, fatigue), and QoL (daily activity complaints, cancer-related QoL) with PI in cancer survivors.

## 2. Materials and Methods

### 2.1. Procedure and Participant Recruitment

This multicenter cross-sectional study follows Dutch law and the principles of the Declaration of Helsinki. Cancer survivors were recruited by convenience sampling from various randomly selected treatment centers, mainly private practices, which specialized in oncological rehabilitation across the Netherlands between December 2017 and March 2020. Participants with any type of cancer, at any stage of rehabilitation, and of all sexes were included in this study when fulfilling the following criteria:

- Being a cancer survivor is defined by The European Organization of Research and Treatment of Cancer (EORTC) Survivorship Task Force as: ‘any individual who has been diagnosed with cancer, has completed his or her primary treatment (with the exception of maintenance therapy), and has no evidence of active disease [35].
- Being ≥18 years old.
- Being native Dutch speakers and readers.

Participants were excluded if they were diagnosed with metastasized cancer or any comorbidity such as a medical or psychiatric disease, precluding them from understanding and filling in the questionnaires. All eligible participants provided written informed consent prior to enrollment. Subsequently, they received an information letter and a standard set of questionnaires to fill out on paper within three days.

### 2.2. Outcome Measures

#### 2.2.1. Sociodemographic Data

Information on age, sex, cancer type, cancer history, medical treatment, comorbidities, education, and current work status were collected. 

#### 2.2.2. Perceived Injustice

The IEQ provided an inventory for the patient’s frequency of 12 different pain-related thoughts regarding the unfairness of their injury on a 5-point scale ranging from 0 (never) to 4 (all the time) [16]. A higher score represented a higher level of PI. The total score ranged from 0 to 48 [36]. The Dutch version of IEQ generated data with good reliability (ICC = 0.86–0.87) [37] and validity [36].

#### 2.2.3. Pain-Intensity

Pain intensity was assessed using a Numeric Pain Rating Scale (NPRS) [38,39,40]. This ordinal scale ranged from 0 (no pain) to 10 (most imaginable pain) [38,39,40]. The participant needed to circle the number that represented their pain during the past week [38,39,40]. The NPRS generates data that shows good reliability and validity [39]. This scale is applicable to diverse pain populations [39].

#### 2.2.4. Fatigue

Fatigue was assessed by the Multidimensional Fatigue Index (MFI) [41]. The MFI is a 20-item self-report instrument divided into five domains: general fatigue, physical fatigue, reduction in activity, reduction in motivation, and cognitive fatigue [41]. All questions are scored on a scale ranging from 1 (disagree) to 5 (totally agree), with a total score that ranges between 20 and 100 [41]. Higher scores on the MFI indicate a higher level of fatigue [42]. The MFI generates data that are reliable and valid when measuring fatigue in Dutch cancer patients [42].

#### 2.2.5. Patient-Specific Complaints

The Patient-Specific Complaints instrument (PSC) was used to assess problems during daily activities [43,44]. The degree to which a maximum of the 5 most important complaints are experienced is reported on a visual analog scale (VAS), ranging from 0 (no complaints) to 100 (impossible to perform the activity) [43,44]. A final score is calculated using the mean of all described activities on the PSC. The validity and responsiveness of the data generated using the Dutch PSC are moderate [45].

#### 2.2.6. Quality of Life

The European Organization for Research and Treatment of Cancer Quality of Life Questionnaire–C30 (EORTC-QLQ-C30) is a 30-item instrument that was developed for people with cancer and examines several aspects of health-related QoL in cancer patients. Only items 29 and 30 of this questionnaire are needed to assess global health status/QoL. These two questions have a 7-point scale ranging from 1 (very poor) to 7 (excellent). A higher total score represents a higher degree of QoL [46,47]. The EORTC QLQ-C30 has been translated and validated in Dutch, is widely used in cancer studies, and shows acceptable psychometric properties [46,47]. 

### 2.3. Statistical Analyses

SPSS version 28.0 (SPSS Inc., Chicago, IL, USA) was used for statistical analyses. Descriptive statistics were executed for personal characteristics, symptoms, and QoL. The Kolmogorov–Smirnov test was used to examine whether the variable residuals were normally distributed. Frequencies were reported as the number of cases with a corresponding percentage. Normally distributed variables are expressed as the mean ± standard deviation (SD), with not normally distributed variables as the median and interquartile range (IQR). Outliers and extreme values were detected [48]. 

Sullivan et al. (2008) defined that the clinically relevant cut-off score corresponds to the 75th percentile of the distribution of IEQ scores in clinical samples with chronic pain [16]. Based on this hypothesis and knowing that cancer survivors appear to have a high prevalence of chronic pain [28], a clinically relevant cut-off score on the IEQ score was generated for cancer survivors to determine the 75th percentile of the IEQ scores. 

Univariate linear regressions were performed with the IEQ scores as the dependent variable and personal characteristics, symptoms, and QoL as the independent variables. Categorical variables were transformed into dummy variables. If only one category was possible within one person, a reference category was chosen. If a participant fit into different categories, all categories were maintained as dummy variables. A significance level of *p* < 0.20 for the univariate regression effects was used to include the independent variable in the multivariate linear regression analysis. For the categorical variables, the category with the lowest significance level was used for that variable. The multivariate linear regression was performed with a backward elimination procedure. For this, the independent variable with the highest *p*-value was removed until all remaining variables had a significance value of *p* < 0.05 for their effect. All assumptions were checked for the final model. Missing data were handled pairwise, and the statistical significance level was set at *p* < 0.05 (two-tailed) for all analyses. 

## 3. Results

### 3.1. Descriptives of Personal Characteristics, Symptoms, and Quality of Life

A total of 121 participants were included in this study. The descriptives are presented in Table 1. The mean age of the study participants was 59.0 ± 13.3 years. Thirty-two participants (26.4%) were male, and 89 participants (73.6%) were female. The most common type of cancer was breast cancer, with a total of 57 cases (47.1%). Most of the participants underwent surgery (69.4%), chemotherapy (60.3%), and/or radiotherapy (59.5%) as one of their medical treatments.

The median of the IEQ score was 13.0 (95% CI: 9.0–20.0). The NPRS showed a median score of 1.5 (95% CI: 0.0–3.0) and the MFI 61.0 (95% CI: 47.0–71.8). The median score of the PSC was 58.0 and 56.0 (95% CI: 50.0–63.0) for the EORTC-QLQ-C30 (subscale QoL). 

There was no multicollinearity available between the different variables included in the further analyses (Table 2).

### 3.2. Clinical Cut-Off for Cancer Survivors 

A clinical cut-off score of 20 was identified for cancer survivors by taking the 75th percentile of the IEQ (Table 3) [16]. Based on this new clinical cut-off value for cancer survivors, 30 cancer survivors experienced clinically relevant levels of PI, and 89 cancer survivors did not experience clinically relevant levels of PI (Table 1). 

### 3.3. The Relationships with Perceived Injustice 

Univariate linear regression analyses of personal characteristics with IEQ scores demonstrated a significant indirect effect of 3.321 with chemotherapy (*p* = 0.029; 95% CI: 0.346 to 6.295). Based on these results, it was revealed that hormonal therapy or immunotherapy is associated with lower levels of PI compared to chemotherapy, radiotherapy, and surgery, predicting higher levels of injustice. No significant effects were found for sex (B = 1.520; *p* = 0.363; 95% CI: −1.779–4.820), age (B = −0.094; *p* = 0.103; 95% CI: −0.208–0.019), or the type of cancer (B = 3.982; *p* = 0.133; 95% CI: −1.226–9.190) with IEQ scores. Interestingly, every other type of cancer predicted higher levels of PI compared to breast cancer. On the other hand, all indirect effects of the symptoms and QoL with IEQ scores were significant (Table 4). Positive indirect significant effects were found for pain intensity (B = 0.863; *p* = 0.016; 95% CI: 0.161–1.566), daily activity complaints (B = 0.067 [0.008 to 0.127], *p* = 0.027), and fatigue (B = 0.204; *p* < 0.001; 95% CI: 0.124–0.284). This indicates that higher scores on pain intensity, daily activity complaints, and fatigue are related to higher IEQ scores. By contrast, a negative indirect significant effect of −0.167 was found for QoL (*p* < 0.001; 95% CI: −0.252–−0.083). In other words, higher scores of QoL are related to lower IEQ scores.

Based on the results of the univariate analyses, all variables, except sex, were included in the multivariate model (*p* < 0.20) in the following decreasing order of significance: MFI, EORTC-QLQ-C30, NPRS, PSC, type of treatment, age, and cancer type. Following the backward elimination, only fatigue (Wald-statistic = 30.260; *p* < 0.001 ***) and age (Wald-statistic = 5.868; *p* = 0.015 **) were found to have a statistically significant direct model effect in the final multivariate model (Table 5).

All assumptions (linearity, homoscedasticity, normality of the residuals, and independence) were met for the final model.

## 4. Discussion

This is the first study to investigate a clinically relevant cut-off score for PI and explore the relationship between personal characteristics (sex, age, type of cancer, treatment type), symptoms (pain intensity, fatigue), and QoL (daily activity complaints, cancer-related QoL) with PI in cancer survivors. A clinically relevant cut-off score of 20 for PI was identified and measured with the IEQ. The regression models demonstrated significant direct effects with age and fatigue, with higher scores for fatigue and lower age being related to higher levels of PI. Indirect significant effects were found for chemotherapy and for all symptoms and QoL outcomes. These results indicate that higher scores on pain intensity, daily activity complaints, and fatigue are related to higher PI levels, but higher scores on QoL are related to lower PI levels. This is in line with previous research in different populations looking into the relationship of PI in people with pain [18,19]. 

In the final model, only age and fatigue appeared to significantly affect PI in cancer survivors. These findings indicate that fatigue and age are important direct associates of PI. To the best of our knowledge, associations between age and PI have never been explored in cancer survivors before. No significance was found for an indirect association between age and PI. However, age was found to be directly related to PI based on the multivariate model, which also contained fatigue as an independent factor. Therefore, there also seems to be a link between age and fatigue, which is not surprising, considering that the prevalence of cancer-related fatigue is higher in older people compared to younger people [22]. Due to degenerative changes in organs during aging, older people are more susceptible to the side effects induced by cancer therapy, resulting in more severe comorbidities [49]. More severe fatigue in older people can also be caused by a decrease in the number and function of energy-producing mitochondria [49]. However, this study also demonstrated that younger cancer survivors experience higher levels of PI. This seems logical, considering that QoL is negatively impacted in the cancer survival stage [50]. Although this can be highly inter-individually dependent, it can be assumed that younger people perceive the impact on their independence, employment, financial security, and identity as a bigger loss in life compared to older cancer survivors [51]. This loss in multiple life domains can then be perceived as unjust [52,53]. As cancer can be considered an aging disease, younger people affected by cancer can more easily perceive their disease as unfair. Unfortunately, age is not an adaptable factor, but should be considered as a possible influencing factor for PI in cancer survivors. 

This study also indicates that more fatigue is seen together with higher levels of PI in cancer survivors. Previous research has not investigated PI and fatigue, except in one paper in which PI was found to be significant in all direct and indirect relationships with fatigue and sleep disturbances in breast cancer survivors [11]. It is known that individuals with PI express more painful behavior (i.e., the more intense pain communication of their suffering and losses). This pain behavior increases the likelihood of being prescribed opioids [54]. This is worrying since long-term opioid use decreases sleep quality and daytime function [55]. The use of sedatives also reduces sleep quality in some cancer survivors [55,56,57], leading to daytime fatigue and abnormal sleep patterns [55,58]. Moreover, sleep disturbances can occur when healthy employees experience a sense of injustice in their work [59]. Additionally, cancer survivors who have chemotherapy are at risk for developing cancer-related fatigue [22]. This is interesting because chemotherapy is also indirectly associated with PI in our study. 

Even though pain intensity and daily activity complaints/cancer-related QoL were not significantly related to PI based on the multivariate model, the results of the univariate analyses still suggest the possibility of these factors being (indirectly) associated with PI in cancer survivors. Correlation statistics also confirm this assumption, as there is a significant correlation between PI and these factors. Previous research found the same positive association between pain intensity and the level of PI in different populations, such as chronic pain [13,25,36,37,54,60,61,62,63,64,65,66,67,68,69,70], low back pain [66,71,72], traumatic injuries [67,73,74,75,76,77], menstrual pain [78], whiplash injury [26,52,79], spinal cord injury (SCI) [80], arthritis and fibromyalgia [27,81], and breast cancer survivors [9,11]. As mentioned before, the increased pain behavior seen in people with PI results in a more intense feeling of suffering and loss [82]. In breast cancer survivors, higher PI scores were related to lower QoL and PI rather than pain catastrophizing, mediating the relationship between pain and QoL [9]. This shows that PI partially influences QoL, which is not surprising since PI can arise in response to experiences characterized by suffering and losses in multiple life domains [52,53], including a loss of independence, employment, financial security, and the loss of identity [51]. The vast majority of studies assessing PI investigated PI as a predictor of adverse physical and mental health outcomes associated with pain, such as long-term disabilities and poor rehabilitation outcomes [12].

Besides relationships with PI, a clinically relevant cut-off of 20 on the IEQ was defined. Previously, Sullivan et al. (2008) defined a clinically relevant cut-off score of 30 on the IEQ in individuals who had sustained musculoskeletal injuries [16]. He described that the clinically relevant cut-off score corresponds to the 75th percentile of the distribution of IEQ scores in clinical samples of chronic pain patients [16]. A second frequently used cut-off score for PI of 19 was defined by Scott et al. (2013) in whiplash patients. This cut-off was defined by a receiver-operating characteristic curve according to the clinical risk outcome variable of return to work [17]. Two additional cut-offs for PI were identified by Scott et al. (2013) with two other risk outcomes: pain severity with a cut-off of 18 and narcotic use with a cut-off of 20 [17], which is similar to the cut-off defined in this study. It is seen that cancer survivors demonstrate a significantly higher opioid prescription rate compared to controls without a prior cancer diagnosis [83,84]. This may be an indication of the corresponding cut-off value between the clinical cut-off calculated in cancer survivors and the cut-off identified for narcotic use in individuals following whiplash injury. 

### 4.1. Clinical Implications

The definition of a population-specific cut-off score for the general cancer survival population makes the identification of clinically relevant levels of PI when measured on the IEQ, possible in clinical practice and research. As mentioned before, cancer survivors have significantly higher opioid use than non-cancer patients [83,84], which is also seen in people with higher levels of PI [82]. PI is associated with increased opioid prescription [82] and prospectively predicted opioid use at 1-year of follow-up [17]. Pharmacological treatment approaches should thus be handled with care in cancer survivors with PI and/or fatigue, and new non-pharmacological treatment options should be considered. 

Up until now, the literature has suggested that the use of cognitive-behavioral interventions, pain acceptance [12], and educational interventions can encourage and reassure patients toward activity re-engagement [62] for the management of PI. This is in line with the current non-pharmacological management of cancer-related fatigue, including the education of the patient and family, psychosocial interventions (i.e., behavioral therapy, psychotherapy, support groups, changing coping strategies, relaxation, energy conservation, and stress management), and physical activity including aerobic and resistance training [85]. Cognitive-behavioral therapy has the strongest effect on fatigue after insomnia in cancer survivors [86]. Moreover, it also improves functional health and, thus, improves QoL as well [86]. Recently, a practical guideline for addressing PI in cancer survivors has become available [8], including cognitive-behavioral therapy. However, further high-quality longitudinal randomized clinical trials are needed for the experimental testing of this proposed best-evidence treatment approach. 

### 4.2. Study Limitations and Strenghts

This is the first study focusing on PI in the general cancer survivor population defining a population-specific cut-off. Further research is needed to validate this cut-off for further use in clinical research and practice. Moreover, this is the first exploration of the association between personal cancer-related factors (type of cancer and treatment) with PI. Due to the unavailability of data, it was not possible to consider the perception of PI (e.g., “Why do you think your life will never be the same again?”), the time since cancer diagnosis, the specific type/dose of surgery, chemotherapy, or radiotherapy, as well as the order of the curative treatment in the analyses, which all could have an impact on the level of PI. Moreover, known important measures such as depression, anxiety, and pain acceptance are missing as possible relevant predictors of PI in cancer survivors.

Since the recruitment was based on the patient’s willingness to participate, selection bias could be possible. Moreover, an under- or overrepresentation of groups within the sample is possible due to the use of convenience sampling, making it difficult to generalize the results of this study to all cancer survivors. Considering the explanatory character of this study, no power calculation was conducted a priori. Additionally, with the cross-sectional design of this study, no claims on causality between PI and personal factors, symptoms, and QoL can be made. Still, this exploratory study calls for future longitudinal research to further examine these relationships.

## 5. Conclusions

A clinical cut-off of 20 for the score of the IEQ was defined for the cancer survivor population. Significant direct associations were found for PI with age and fatigue PI in cancer survivors. However, associations with PI in cancer survivors should be further examined to explore causal interactions.

## Figures and Tables

**Table 1:** Descriptives of personal characteristics, symptoms, and quality of life in Dutch cancer survivors (n = 121).

		Number of Participants (%)
Sex	Female	89 (73.6%)
	Male	32 (26.4%)
Perceived injustice	Present	30 (25.2%)
	Absent	89 (74.8%)
Type of cancer	Breast cancer	57 (47.1%)
	Lung cancer	12 (9.9%)
	Colon cancer	11 (9.1%)
	Lymphoma	6 (5.0%)
	Other types of cancer	35 (28.9%)
Treatment type	Chemotherapy	73 (60.3%)
	Radiotherapy	72 (59.5%)
	Surgery	84 (69.4%)
	Immunotherapy	5 (4.1%)
	Hormonal therapy	38 (31.4%)
		**Mean ± SD/median (IQR)**
Age (years)		59.0 ± 13.3
Perceived injustice	IEQ (n = 119)	13.0 (9.0–20.0)
Symptoms	NPRS (n = 114)	1.5 (0.0–3.0)
	MFI (n = 120)	61.0 (47.0–71.8)
Quality of Life	PSC (n = 117)	58.0 (35.0–69.5)
	EORTC-QLQ-C30 (n = 113)	56.0 (50.0–63.0)

Abbreviation(s): EORTC-QLQ-C30 = European Organization for Research and Treatment of Cancer Quality of Life Questionnaire–C30; IEQ = Injustice Experience Questionnaire; IQR = Interquartile Range; MFI = Multidimensional Fatigue Index; N = number; NPRS = Numeric Pain Rating Scale; PSC = Patient Specific Complaints; QoL = Quality of Life; SD = standard deviation.

**Table 2:** Correlations between all variables in Dutch cancer survivors.

	Sex	Age	Chemotherapy	Radiotherapy	Surgery	Immunotherapy	Hormonal Therapy	Breast Cancer	Lung Cancer	Colon Cancer	Lymphoma	Other types of Cancer	IEQ	NPRS	MFI	PSC
Age	−0.226 *															
Chemotherapy	−0.065	−0.145														
Radiotherapy	0.269 **	−0.039	0.054													
Surgery	0.212 *	−0.102	−0.098	0.037												
Immunotherapy	0.124	−0.107	0.083	0.002	0.048											
Hormonal therapy	0.325 **	−0.131	0.148	0.304 **	0.140	0.038										
Breast cancer	0.566 **	−0.139	0.122	0.374 **	0.303 **	0.137	0.646 **									
Lung cancer	−0.365 **	0.121	−0.014	−0.121	−0.080	−0.069	−0.225 *	−0.313 **								
Colon cancer	−0.201 *	0.034	−0.037	−0.091	0.147	−0.066	−0.214 *	−0.298 **	−0.105							
Lymphoma	−0.253 **	0.097	0.027 *	−0.156	−0.373 **	−0.051	−0.168	−0.234 **	−0.082	−0.078						
Other types of cancer	−0.125	0.010	−0.207 *	−0.196 *	−0.184 *	−0.037	−0.344 **	−0.590 **	−0.207 *	−0.198 *	−0.155					
IEQ	0.084	−0.151	0.170	0.024	0.065	−0.139	−0.046	−0.174	0.057	0.101	0.054	0.063				
NPRS	0.070	−0.162	0.065	−0.028	−0.096	−0.090	0.152	0.030	−0.012	−0.012	0.029	−0.033	0.226 *			
MFI	−0.150	−0.036	0.049	−0.110	0.058	−0.127	−0.049	−0.210 *	0.144	0.094	−0.039	0.020	0.423 **	0.292 **		
PSC	−0.174	0.040	0.016	−0.174	0.090	−0.087	−0.158	−0.275 **	0.270 **	0.076	0.123	0.105	0.206 *	0.160	0.526 **	
EORTC-QLQ-C30	0.225 *	0.142	0.047	0.151	0.059	0.144	0.078	0.303 **	−0.271 **	−0.231 *	−0.056	0.007	−0.353 **	−0.378 **	−0.681 **	−0.405 **

Significant correlations are indicated in bold and statistical significance level with asterisks (* *p* < 0.05; ** *p* < 0.01) and color (the darker, the higher the correlation). Abbreviation(s): EORTC-QLQ-C30 = European Organization for Research and Treatment of Cancer Quality of Life Questionnaire–C30; IEQ = Injustice Experience Questionnaire; MFI = Multidimensional Fatigue Index; NPRS = Numeric Pain Rating Scale; PSC = Patient Specific Complaints; QoL = quality of life.

**Table 3:** Number of Dutch cancer survivors per percentile of the IEQ scores (n = 119).

	**P0**	**P5**	**P10**	**P15**	**P20**	**P25**	**P30**	**P35**	**P40**	**P45**	**P50**	**P60**	**P65**	**P70**	**P75**	**P80**	**P85**	**P90**	**P95**	**P100**
N=	0	3	5	6	7	9	10	11	12	12	13	16	17	18	**20**	21	23	25	28	37

Abbreviation(s): IEQ = Injustice Experienced Questionnaire; N = number; P = percentile. Percentile 75 is indicated in bold.

**Table 4:** Univariate linear regression analyses with IEQ scores as the dependent variable and personal characteristics, symptoms, and quality of life as independent variables in Dutch cancer survivors (n = 121).

	*p*-Value (F)	B	SE B	Stand. b	*p*-Value (t)	95% CI
Personal characteristic						
*Constant* Sex (Ref: male)	0.363	11.835 1.520	2.989 1.666	0.084	0.363	5.915 to 17.754 −1.779 to 4.820
*Constant* Age	0.103	20.090 −0.094	3.475 0.057	−0.151	**0.103**	13.207 to 26.972 −0.208 to 0.019
*Constant* Type of cancer (Ref: breast cancer) Lung cancer Colon cancer Lymphoma Other types of cancer	0.413	13.018 2.815 3.982 3.315 1.733	1.065 2.536 2.629 3.424 1.733	0.107 0.145 0.091 0.128	0.269 **0.133** 0.335 **0.197**	10.908 to 15.128 −2.208 to 7.839 −1.226 to 9.190 −3.468 to 10.099 −1.187 to 5.680
*Constant* Treatment type Chemotherapy Radiotherapy Surgery Hormonal therapy Immunotherapy	0.148	13.938 3.321 0.625 1.717 −1.651 −6.247	5.563 1.501 1.543 1.604 1.657 3.611	0.205 0.039 0.099 −0.996 −1.730	0.029 * 0.696 0.287 0.321 0.086	2.917 to 24.959 0.346 to 6.295 −2.432 to 3.682 −1.461 to 4.896 −4.935 to 1.632 −13.401 to 0.906
**Symptoms**						
*Constant* NPRS (/10)	0.016	13.421 0.863	1.029 0.354	0.226	0.016 *	10.382 to 14.460 0.161 to 1.566
*Constant* MFI (/100)	<0.001	2.506 0.204	2.478 0.040	0.423	<0.001 ***	−2.401 to 7.414 0.124 to 0.284
**Quality of life**						
*Constant* PSC (/100)	0.027	10.870 0.067	1.708 0.030	0.206	0.027 *	7.487 to 14.253 0.008 to 0.127
*Constant* EORTC-QLQ-C30 (subscale QoL) (/100)	<0.001	25.416 −0.167	2.938 0.043	−0.353	<0.001 ***	19.594 to 31.239 −0.252 to −0.083

Variables with significance level *p* < 0.20 to be included in the multivariate model are indicated in bold. Statistical significance level: * *p* < 0.05; *** *p* < 0.001. Abbreviation(s): CI = Confidence Interval; EORTC-QLQ-C30 = European Organization for Research and Treatment of Cancer Quality of Life Questionnaire–C30; IEQ = Injustice Experience Questionnaire; MFI = Multidimensional Fatigue Index; NPRS = Numeric Pain Rating Scale; PSC = Patient Specific Complaints; QoL = quality of life; Ref = reference; SE = standard error; Stand. = standardized.

**Table 5:** Multivariate linear regression analysis with IEQ scores as the dependent variable and fatigue and age as direct significant associates in Dutch cancer survivors (n = 121).

	B	SE B	Stand. b	*p*-Value	95% CI	Adj. R^2^
*Constant*	7.583	4.014	1.889	0.061	−0.369 to 15.534	18.7%
MFI	0.205	0.040	−1.633	<0.001	0.125 to 0.285	
Age	−0.086	0.053	5.070	0.105	−0.191 to 0.018	

Abbreviation(s): Adj. = adjusted; CI = Confidence Interval; MFI = Multidimensional Fatigue Index; SE = standard error; Stand. = standardized.

## Data Availability

The data that support the findings of this study are available upon reasonable request to the corresponding author.
